# A Prospective Study on the Impact of Clinical Factors and Adjusted Triple D System for Success Rate of ESWL

**DOI:** 10.3390/medicina59101827

**Published:** 2023-10-13

**Authors:** Marius Snicorius, Mingailė Drevinskaitė, Marius Miglinas, Albertas Čekauskas, Vidita Urbonienė, Rimantė Bandzevičiūtė, Justinas Čeponkus, Valdas Šablinskas, Arunas Želvys

**Affiliations:** 1 Institute of Clinical Medicine, Faculty of Medicine, Vilnius University, 03225 Vilnius, Lithuania; 2 Institute of Chemical Physics, Faculty of Physics, Vilnius University, 10222 Vilnius, Lithuania

**Keywords:** urolithiasis, extracorporeal shock wave lithotripsy, triple D score, renal stones, computed tomography, stone density, stone volume, stone free rate

## Abstract

*Objective*: Our study aimed to evaluate the success rate of ESWL and identify relevant treatment-specific factors affecting treatment outcomes, as well as to assess the accuracy of the updated Triple D scoring system and compare it with older systems. *Material and Methods*: A prospective study of 71 patients who received ESWL treatment for renal stones that were 5–15 mm in size was completed. The patient having no residual stones or residual stones lesser than 4 mm after ESWL was identified as a treatment success. Univariate and multivariate logistic regression and ROC curves were used to identify important factors for treatment outcomes. *Results*: Successful treatment was achieved for 66.2% of patients. The stone volume (SV), mean stone density (MD), and delivered power to the stone volume unit ratio (SMLI/SV) were defined as the most critical factors influencing ESWL success. An updated Triple D score system with a, SMLI/SV ratio could be an alternative to older systems and reach an even higher accuracy. A limitation of this study is the limited sample size due to the COVID-19 pandemic. *Conclusions*: Our results show that the three factors that most influence the success of ESWL are the stone size, mean stone density, and SMLI/SV ratio. Based on this, we present a simple updated triple D score system to predict ESWL success, which could be implemented in future clinical practice.

## 1. Introduction

The global patterns of urolithiasis are changing, as the prevalence of urinary stones has increased in developed and developing countries over the past few years [1]. Urinary tract stones affect 1 in 10 people at least once in a lifetime worldwide; in 2% of people, the disease is recurrent [2]. Endourological procedures, such as ureteroscopy and percutaneous nephrolithotomy, are gaining popularity. Consequently, extracorporeal shockwave lithotripsy (ESWL) has lost its place as a primary treatment choice despite its proven efficiency [3,4]. According to the urological guidelines, ESWL remains an option for treating renal stones that are sized up to 20 mm [3]. On the technical side, the efficiency of ESWL can be increased by optimizing the sequence of shock waves, dose escalation of the shock wave energy, and the number (2000–4000) and frequency (1.0–1.5 Hz) of the shock waves [5]. However, proper patient selection is crucial for good treatment outcomes. Many parameters affecting stone-free rates (SFR) after ESWL have been determined: stone size and location [1,6,7], stone attenuation values on computed tomography (CT) [8], skin-to-stone distance (SSD), and others [9,10].

Various nomograms were constructed for ESWL treatment outcome prediction. However, most of them are often too complex to calculate in the clinical setting. In 2015, Tran et al. [11] presented a simplified Triple D scoring system to check the most suitable patients for ESWL, which is constructed by three parameters calculated in computed tomography: skin-to-stone distance, stone volume, and stone density. Its clinical accuracy has been externally validated in different retrospective studies [12,13]. A few years later, Ichiyanagi et al. [14] reinforced the Triple D score system with the Quadruple D score system by adding the lower calyx stone as an additional parameter. According to the results of the study, the accuracy of the Quadruple D score system was significantly higher than that of the Triple D system [14].

Our study aimed to evaluate the clinical efficacy of the Triple and Quadruple D score systems for patients with 5–15 mm stones. In addition, we proposed a novel factor, which could contribute to an even more accurate prediction of the success of the treatment, and upgrade previously described scoring systems.

## 2. Materials and Methods

A total of 146 patients were included in this prospective study. This study was conducted according to the guidelines of the Declaration of Helsinki and approved by the Vilnius regional biomedical research ethics committee (approval number No. 2019/3-1108-606 on 26 March 2019). Our study included patients treated with ESWL procedures for renal stones from 2019 March to 2022 March, mainly during the COVID-19 pandemic, at the Urological Tertiary Centre of Vilnius University Santaros clinics in Vilnius, Lithuania. Out of 146, only 71 patients met the study inclusion criteria: accessible computed tomography scans before and after ESWL treatment and a stone size ranging from 5 to 15 mm. The exclusion criteria, such as pregnancy, active urinary infection, uncorrected coagulopathy, and patients using anticoagulants on the day of ESWL, were absolute contraindications to ESWL. Patients who did not have NCCT before the treatment, whose maximum stone diameter was greater than 15 mm, or whose multiple stones were on the same side were excluded from the study.

All patients were treated under the same protocol in the supine position using a Storz Modulith SLK lithotripsy machine using ultrasound guidance (Storz Medical, Tuttlingen, Germany) without anesthesia. Three different urologists performed ESWL procedures. During the ESWL procedure, up to 3000 shock waves were delivered to the stone with a gradual power increase of up to 85 mJ, maintaining a frequency of 1.5 Hz during all of the sessions. The total amount of energy applied to the stone was calculated using the Storz Medical Lithotripsy Index (SMLI). Patients would receive a maximum of two ESWL procedures for urolithiasis treatment with a one-month waiting period between them if the fragmentations were incomplete after the first procedure. Some patients had only one ESWL due to renal colic, which resulted in a stenting procedure and then endoscopic removal of the stone. During the follow-up period, the treatment efficacy was evaluated by using ultrasound and computed tomography. The final verification of the patient’s stone status was done three months after the last ESWL procedure with ultrasound and CT. This study defined successful treatment as whether patients were diagnosed with residual stone fragments whose diameter was less than 4 mm or no residual fragments. If the patient had residual pieces over 4 mm or required a ureteral stenting procedure and additional endourological stone treatment—the clinical situation was defined as treatment failure.

Variables such as SSD, maximum stone diameter (MSD), stone volume (SV), mean stone density in Hounsfield units (MD), and the highest Hounsfield unit score were obtained pre-operatively from CT images. SSD was calculated as the average distance from the skin to the surface of the targeted stone at 0°, 45°, and 90° angles on CT. MSD was measured in the sagittal, transversal, and coronal body planes. SV was calculated using the formula: SV = l × w × d × π × 0.167, where l is length, w is width, d is depth, and π = 3.14159. For the measurement of the stone density, all three body planes were defined for each stone. In each plane, an area of interest smaller than the stone was depicted where stone density was measured, and the mean value was calculated. The total amount of energy applied to the stone was computed using the SMLI. Furthermore, the Triple D Score was calculated for patients based on the number of parameters that fell below the cut-off values. The cut-off values of <150 mm^3^ for stone volume, <120 mm for SSD, and <600 Hounsfield unit (HU) for stone density were established as described by Tran et al. [1]. The Quadruple D scoring system was defined as a triple D score combined with the stone location in the kidney. The location was allocated 0 points if the stone was placed at the lower calyx and 1 point if the stone was located in a different part of the kidney collective system. The score would range from 0 (worst) to 3 (best) points and from 0 (worst) to 4 (best) points in the Triple D and Quadruple D scoring system, respectively.

All statistical tests were performed using SPSS software 26.0 (IBM Corp., Armonk, NY, USA). A *p* value of <0.05 was considered statistically significant. Continuous variables are presented as means with standard deviations (SD). Data for categorical variables are presented as frequencies and percentages. Continuous variables were checked for normal distribution by the Shapiro–Wilk test and compared by the t-test when normally distributed or the Mann–Whitney U test for non-normally distributed variables. Pearson’s χ2 and Fisher’s exact tests were used to compare categorical variables, as appropriate. To identify predictors for ESWL success, univariate and multivariate logistic regression analysis was performed, where odds ratios (OR) and 95% confidence intervals (CI) were calculated. Receiver operating characteristic (ROC) curves were generated, and areas under the curves (AUC) were analyzed to compare the predictive power of different factors and score systems for treatment success.

## 3. Results

A total of 71 patients were included in the final study analysis. A total of 44 patients (62%) received two ESWL procedures and 27 patients (38%) received only one ESWL procedure due to total stone disintegration after the first intervention or the patient suffering from renal colic and urinary tract obstruction and ureteral stenting being performed as a result. After stenting, no further ESWL procedures were performed. Instead, the patient underwent endourologic urolithiasis treatment.

Overall, successful treatment was achieved for 47 (66.2%) patients. Detailed treatment efficacy results: 18 (25,4%) patients were totally stone free, 29 (40.8%) had residual fragments <4 mm, and 24 (33.8%) patients ended up with treatment failure as residual fragments were >4 mm or endoscopic treatment was performed due to renal colic. Out of the 27 patients who received only one ESWL procedure, complete stone-free status was achieved in 6 (22.2%) cases, the residual fragments were <4 mm in 10 (37%) cases, and there was treatment failure in 11 (40.7%) cases. Better results were achieved in the 44-patient group who received two ESWL procedures: complete stone-free status was achieved in 12 (27.3%) cases, the residual fragments were <4 mm in 19 (43.3%) cases, and treatment failure was seen in 13 (29.5%) cases. In conclusion, successful treatment was seen in 59.3% of patients after one ESWL and in 70.5% of cases when two ESWL procedures were completed (*p* = 0.439). Ureteral stenting or adjunctive endoscopic treatment for stone removal was performed for 22 (31%) patients. A total of 49 patients were successfully treated only with ESWL procedures.

A stone location in the lower calyx was observed for 45 (63.4%) patients, while 26 (36.6%) patients‘ stones were localized in the renal pelvis or a different calyx. Treatment was successful for 64.2% of the patients when a stone was in their lower calyx and for 69.2% when a stone was observed in a different part of the collecting system (*p* = 0.443). However, having a stone in the lower calyx was a clinically significant factor for total stone disintegration, and removal of the stone after the ESWL procedures. Out of 45 patients with lower calyx stones, only 7 (15.6%) achieved a totally stone-free status, while differently localized stones were disintegrated and totally removed in 42.3% of the cases (*p* = 0.022). The location of the stone was not a clinically significant factor for ureteral stenting or adjunctive endoscopic treatment procedures (*p* = 0.300).

Successful treatment (totally stone free or residual fragments <4 mm) was associated with MSD, higher SV, higher MD, and higher SMLI/SV ratios during the procedures (Table 1, all *p* < 0.05). There was no significant difference in age, gender, stone laterality, or stone location between stone-free patients and those who were not.

The mean SV of the stone-free patients following ESWL was 151.34 ± 159.56 versus 284.63 ± 220.67 for those who failed treatment (*p* = 0.005). The MSD had similar differences between the groups: 7.73 ± 2.84 mm versus 10.23 ± 2.88 mm (*p* = 0.001). For MD, the attenuation value was 689.57 ± 261.44 Hounsfield units (HU) for positive outcome patients and 875.08 ± 252.97 HU for patients with treatment failure (*p* = 0.006). The maximum attenuation value was 961.64 ± 369.3 HU for the successfully treated patients’ group and 1191.83 ± 268.18 for the failure group (*p* = 0.009). An average SSD of 97.94 ± 18.01 mm was measured for those who were treated successfully, and an average of 105.88 ± 22.361 mm was noted for those who were not (*p* = 0.11). Finally, the delivered power of the shockwaves during the procedures did not show clinically significant differences between the groups: 318.87 ± 100.79 SMLI versus 293.96 ± 113.63 SMLI (*p* = 0.35). However, when the SMLI/SV ratio was calculated for each group, a difference was noted: 4.02 ± 3.07 versus 1.87 ± 1.58 (*p* = 0.002). Further results of the factor analysis for successful treatment, totally stone-free status, and treatment failure are presented below in Table 2.

During univariate logistic regression, the stone volume, diameter, and SMLI/stone volume ratio outperformed other clinical characteristics and revealed the highest predictive power for ESWL success, where the odd ratios for the stone volume and stone diameter were 0.99 (0.99–1.00) and 0.75 (0.62–0.90), respectively (Table 3, all *p* < 0.05). A tendency was observed for an SSD distance of 0.98 (0.95–1.01), but it did not achieve clinical significance (*p* = 0.115). The SMLI/SV ratio was predictive for a treatment success OR of 1.58 (1.15–2.17) (*p* = 0.005). In the multivariate logistic regression analysis (Table 3), these factors did not remain as statistically significant prognostic factors for treatment success (all *p* > 0.05).

**Figure 1:**
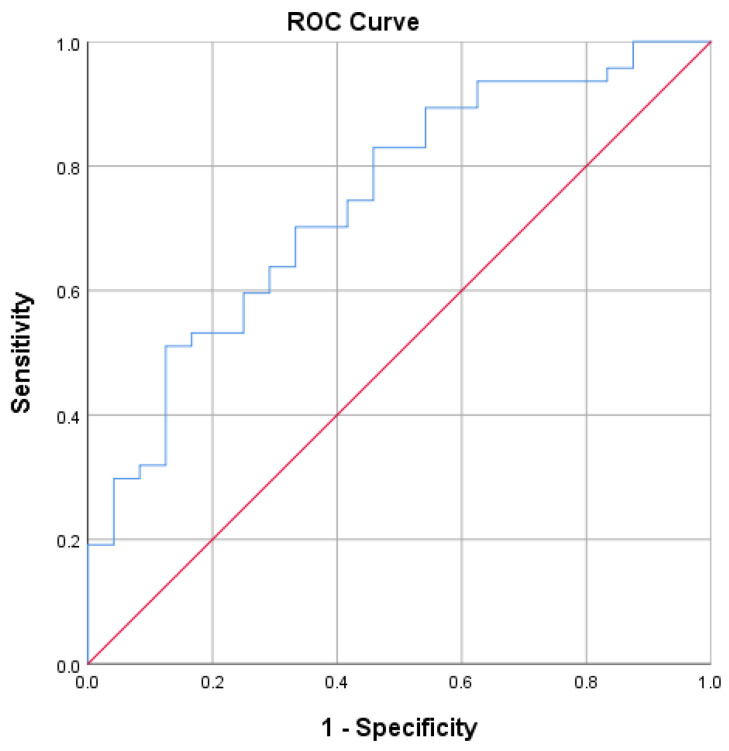
The most crucial factor for extracorporeal shock wave lithotripsy success is the ratio between delivered power to the stone during the procedures and stone volume AUC 0.744 (*p* = 0.001).

ROC curves were generated for each parameter. The area under the curve (AUC) for the SMLI/SV ratio was 0.744 (*p* = 0.001), a sum of SMLI 0.587 (*p* = 0.231), SSD 0.364 (*p* = 0.063), SV 0.730 (*p* = 0.002), MSD 0.740 (*p* = 0.001), and MD 0.688 (*p* = 0.010). The most significant independent predictor for ESWL success was the SMLI/SV ratio (Figure 1), followed by the SV and MSD.

Further ROC analysis revealed that the SMLI/SV ratio is also the most robust predictor of reaching a totally stone-free status after ESWL treatment, with an AUC of 0.741 (*p* = 0.002). The optimal cut-off value for the SMLI/SV ratio is 0.681 (sensitivity 0.94 and 1-specificity 0.71).

Finally, Triple D and Quadruple D scores were calculated for our treated patients. A Triple D score of 0, 1, 2, and 3 correlated with stone-free rates of 33%, 48%, 76.9%, and 92.9%, respectively (Table 4). The Quadruple D score was also calculated for the patients. Additional points were added if a targeted stone was not in the lower calyx. Quadruple D scores of 0, 1, 2, 3, and 4 correlated with stone-free rates of 0%, 50%, 70.8%, 85.7%, and 87.5%, respectively.

**Figure 2:**
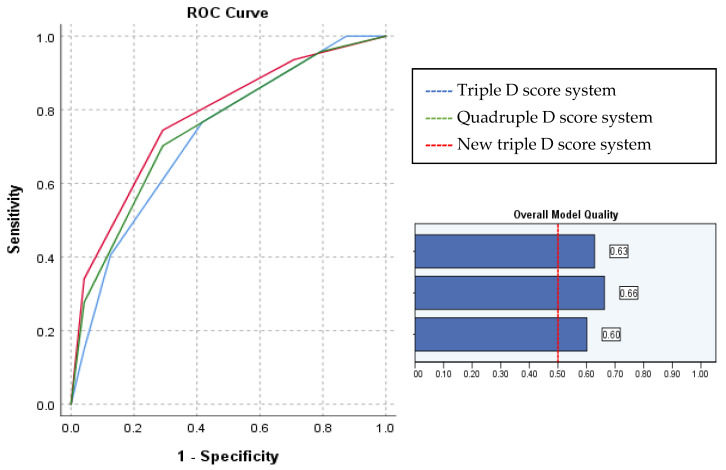
ROC curves for Triple D, Quadruple D, and new Triple D score systems and overall model quality for accurate ESWL treatment success prediction.

According to our results, the SSD was a weaker predictor than the MD and SV for treatment success. Due to these results, we have decided to change SSD with an SMLI/SV ratio over 0.681 as a predictor for the Triple D scoring system. The new triple D score system delivered even better results than the original Triple D or Quadruple D score systems. Generated ROC curves (Figure 2) revealed that the new Triple D system is the most accurate for treatment success prediction, with an AUC of 0.775 (*p* < 0.001). The new Triple D score system of 0, 1, 2, and 3 correlated with successful treatment rates of 30%, 47.4%, 76%, and 94.1%, respectively.

## 4. Discussion

Only a few studies analyzed how the power delivered to the stone volume affects the outcomes of ESWL [15,16]. Both authors defined the SMLI per stone size as a statistically significant factor for predicting ESWL success [15,16]. During our analysis, the power delivered to the stone was measured using the ratio between the SMLI index (shock wave power adjusted by shock wave rate) and the stone volume (SV). Successfully treated patients had a mean SMLI/SV of 4.0 ± 3.08, while the failure group had a lower ratio of 1.87 ± 1.58 (*p* = 0.002). Further ROC analysis revealed that the most crucial factor for ESWL success and a totally stone-free status is an SMLI/SV ratio with an AUC of 0.744 (*p* = 0.001) and an AUC of 0.741 (*p* = 0.002), respectively. Furthermore, with this new parameter instead of the SSD in the Triple D scoring system, outstanding accuracy could be reached for ESWL treatment outcome prediction. These results indicate that the power delivered to a single unit of stone volume is essential for the ESWL outcome and could be used for prediction. Using the optimal cut-off SMLI/SV ratio, it is possible to calculate how much power must be delivered to the stone. If the estimated power score is too high to be achieved during an ESWL treatment procedure, an endourologic treatment path should probably be chosen. The SMLI/SV ratio will differ between machines and treatment centers due to various machine models and treatment protocols or parameters. Still, it is an essential and valuable factor for the treatment outcome. These results indicate that the power delivered to a single unit of stone volume is essential for the ESWL outcome and could be used for prediction.

Previous studies have reported varying ESWL success rates ranging from 46% to 91% [17,18]. In our study, the overall success rate was 66.2%. Such a wide treatment success rate points towards an important role of factors that might influence the overall outcome. Different authors used different definitions of successful and failed outcomes. There is a lack of consensus on defining a successful ESWL treatment. However, we described it as having residual stones <4 mm or having no residual stones at all, and we used this definition when calculating the SFR. Most studies use a stone size <4 mm as a treatment success indicator, but some choose a smaller measurement like <3 mm, as discussed in some studies [19]. For situations with no residual fragments after ESWL, we suggest using the term “totally stone-free” rate (TSFR) to avoid misunderstandings.

The SFR in this study after one ESWL treatment was 59.2%, corresponding well to another study where retreatment was needed in up to 50% of patients [19,20]. This indicates that, to achieve good outcomes, it is necessary to perform more than one ESWL procedure, and patients should be appropriately informed about treatment duration and efficacy.

In our study, the SFRs significantly improved as the number of positive components comprising the Triple and Quadruple D scores increased. Triple D Scores of 0, 1, 2, and 3 correlated with stone-free rates of 33%, 48%, 76.9%, and 92.9%, respectively. The Quadruple D score was also calculated for the patients. Additional points were added if a targeted stone was not in the lower calyx. Quadruple D scores of 0, 1, 2, 3, and 4 were correlated with stone-free rates of 0%, 50%, 70.8%, 85.7%, and 87.5%, respectively. These findings support the successful validation of the implementation of these systems in routine clinical practice and are in line with other studies’ results [11,12,13,14,15]. Furthermore, our study results revealed that SSD is a weaker predictor than other predictors, such as stone volume and stone density, used in scoring systems. Due to these results, we switched SSD with the SMLI/SV ratio, the strongest predictor for successful treatment and totally stone-free status, these having an AUC of 0.744 (*p* = 0.001) and an AUC of 0.741 (*p* = 0.002), respectively. The new Triple D score system of 0, 1, 2, and 3 correlated with successful treatment rates of 30%, 47.4%, 76%, and 94.1% (AUC 0.775; *p* < 0.001).

The effect of SSD on the ESWL success for urinary tract stones is controversial. The SSD predicted the SFR in several studies [21,22], but not always [23]. The cut-off point for a successful treatment in these studies ranges from 9 to 11 cm. In the Triple and Quadruple D score systems, the cut-off value is even higher at 12 cm. However, studies on Asian populations observed that SSD is not a significant predictor for ESWL success because they have thin body volumes compared to Western people [14]. In univariate analyses, the SSD and Body Mass Index (BMI) have been reported as significant predictors for ESWL outcomes [24,25]. In multivariate analyses, either the SSD or BMI is often excluded from the final models for outcome prediction [8,24,25,26], probably due to their correlation. Moderately correlated with the SSD (r = 0.516), the BMI was not different between the groups in the present study. Furthermore, this may be due to the sampling bias resulting from the study design, in which the patients with urinary stones had similar characteristics. Some studies showed a significant relationship between SSD and ESWL success but not between BMI and ESWL. This is probably because, unlike SSD, BMI does not truly reflect central body fat distribution [24]. Due to this reason, in our opinion, BMI should not be used as a predictor for ESWL success.

Stone size, measured on CT as the maximum diameter or stone volume, is a strongly associated factor with a higher SFR after ESWL. The stone volume can be calculated easily, as described in a study by Tran et al. [11], by measuring the anteroposterior, horizontal, and craniocaudal stone diameters. Notably, when the calculated stone volume is compared with the computer-generated 3D stone volume, the correlation coefficient is excellent and reaches 0.9893. The authors found that stone volume strongly predicts ESWL success, with an AUC of 0.775 [11]. Multiple studies have shown that bigger stones have a lower SFR, as the SFR is mainly defined as having fragments smaller than 4 mm [11,12,13,14,20,25]. Stone size as a predictor of the SFR or successful treatment seems to unite most studies on outcomes after ESWL. In our study, univariate logistic regression revealed that the stone volume and diameter of the stone outperformed other clinical characteristics and reached one of the highest predictive powers for ESWL success, where the ORs for stone volume and stone diameter were 0.99 (0.99–1.00) and 0.75 (0.62–0.90). Further ROC analysis achieved outstanding results for stone volume (AUC 0.730; *p* = 0.002) and the maximum diameter of stones (AUC 0.740; *p* = 0.001).

Stone density is another factor that could affect ESWL outcomes and is included in nomograms. Many studies have investigated the relationship between stone density on radiological imaging and its composition and reported that it is possible to predict stone composition from its density [27,28]. This is important because the disintegration depends on the stone’s composition and mineral content. There is still a lack of agreement on how to measure stone density. Techniques are similar but differ, which could contribute to this measurement inconsistency. Muter et al. measured each stone’s mean density in axial and multiplanar reconstruction images at four sites [29]. Other authors calculated the SAV using the mean attenuation of three consistent (area 0.02 cm^2^), non-overlapping regions of interest chosen from stones in bone windows [30]. In the Triple D scoring system, stone density is measured by determining the average HU of an elliptical region of interest over the largest portion of the stone that can be included [11]. Many studies have demonstrated that Stone density has a predictive value for ESWL success. According to other authors, it has different cut-off values, varying between 600 and 1000 HU [11,23,24,25,26,27,28,31]. In our study, successfully treated patients had a stone density of 689.57 ± 261.46 HU, while the treatment failure group had 875.08 ± 252.97 HU (*p* = 0.006). This was also a clinically significant factor in the univariate logistic regression analysis. However, some authors concluded that stone density is an inconsistent factor in predicting ESWL outcomes due to interindividual variability and difficulties in standardizing measurements of stone density [19,21].

More parameters that can be computed using CT scans have been introduced in recent years. The stone heterogeneity index (SHI) is one of them. Patients with ureter calculi were classified into low- and high-SHI groups using their mean SHI and were compared. Multivariate logistic regression analyses revealed that a higher SHI was an independent predictor of one-session success. A smaller stone size and lower MSD were also predictors of success. The authors of this study concluded that SHI is a useful clinical parameter for stone fragility [32]. Another interesting novel predictor of ESWL success is the Variation Coefficient of Stone Density (VCSD) [20]. This new factor represents stone heterogeneity. Investigators compared the predictive powers of treatment success between VCSD and other parameters associated with CT attenuation. In the end, multivariate analysis after categorization by stone location revealed that VCSD was independent and the most significant predictor for shock wave lithotripsy outcomes in kidney and ureteral calculi [33]. A possible weakness of this study is the limited number of participants included because it was completed during the COVID-19 pandemic. Also, the number of patients treated in ESWL facilities per year could be a variable affecting success rates. On the other hand, this study is prospective and has a three-month follow-up period after the treatment to reach actual stone-free rates for the patients. Additionally, all patients included in our study were evaluated with CT before and after treatment, which increases treatment outcome measurement accuracy.

## 5. Conclusions

Stone volume, stone density, and power given to the stone volume are the most crucial factors influencing the success of ESWL. Triple and Quadruple D score systems have been validated for 5–15 mm stones. An updated Triple D scoring system with an SMLI/SV ratio could be an alternative to older systems and achieve even higher accuracy. With this study, we present a simple predictive model for calculating the SFR after ESWL that may contribute to counseling urolithiasis patients. Further studies on a larger scale are needed to validate these results.

## Figures and Tables

**Table 1:** Baseline demographic and clinical characteristics of the study cohort.

Variable	All Patients (N = 71)	Successful Treatment (N = 47)	Treatment Failure (N = 24)	*p*-Value *
Age [years] Mean ± SD	50.85 (13.65)	50.81 (14.40)	50.92 (12.35)	0.975
Gender, N (%): Male Female	46 (64.8%) 25 (35.2%)	34 (72.3%) 13 (27.7%)	12 (50%) 12 (50%)	0.072
BMI Mean ± SD	26.76 (4.34)	25.98 (3.87)	28.18 (4.87)	0.239
Stone location, N (%):				0.443
Other	26 (36.6%)	18 (69.2%)	8 (30.8%)	
Lower calyx	45 (63.4%)	29 (64.2%)	16 (35.6%)	
Max. stone diameter [mm] Mean (± SD)	8.58 (3.07)	7.732 (2.84)	10.229 (2.82)	**0.001**
Stone volume [mm^3^] Mean (± SD)	196.40 (191.74)	151.340 (159.56)	284.625 (220.67)	**0.005**
Mean stone density [HU] (± SD)	752.28 (271.58)	689.57 (261.44)	875.08 (252.97)	**0.006**
Skin to stone distance [mm] Mean (± SD)	100.62 (19.81)	97.94 (18.05)	105.88 (22.36)	0.111
SMLI Mean (± SD)	310.45 (105.61)	293.96 (133.63)	318.87 (100.80)	0.349
SMLI/stone volume ratio Mean (± SD)	3.30 (2.85)	4.02 (3.08)	1.87 (1.58)	**0.002**

BMI—body mass index, HU—Hounsfield units, n—number of patients, SD—standard deviation, SMLI—Storz Medical Lithotripsy Index. * *p* values calculated for comparison of successful and failed treatment cohorts only. Statistically significant *p* values are marked in bold.

**Table 2:** Clinical factors of CT and their impact on successful treatment and stone-free status.

Clinical Factor	Treatment Efficacy	N of Cases	Mean (SD)	*p*-Value	Stone-Free after Treatment	N of Cases	Mean (SD)	*p*-Value
SSD	SFR or fragments <4 mm	47	97.94 18.05	0.111	Totally stone free	18	96.17 15.23	0.273
	Fragments >4 mm	24	105.88 22.36		Residual fragments	53	102.13 21.06	
MSD	SFR or fragments <4 mm	47	7.73 2.84	**0.001**	Totally stone free	18	6.94 2.34	**0.003**
	Fragments >4 mm	24	10.23 2.88		Residual fragments	53	9.13 3.11	
SV	SFR or fragments <4 mm	47	151.34 159.56	**0.005**	Totally stone free	18	113.38 131.56	**0.01**
	Fragments >4 mm	24	284.63 220.67		Residual fragments	53	224.58 201.58	
MD	SFR or fragments <4 mm	47	689.57 261.44	**0.006**	Totally stone free	18	661.33 245.07	0.087
	Fragments >4 mm	24	875.08 252.97		Residual fragments	53	783.17 275.335	
Maximum HU score	SFR or fragments <4 mm	47	961.64 369.30	**0.009**	Totally stone free	18	975.06 365.286	0.375
	Fragments >4 mm	24	1191.83 268.18		Residual fragments	53	1061.3 350.86	
SMLI/SV ratio	SFR or fragments <4 mm	47	4.02 3.08	**0.002**	Totally stone free	18	5.32 3.72	**0.009**
	Fragments >4 mm	24	1.87 1.58		Residual fragments	53	2.60 2.08	

HU—Hounsfield units, N—number of patients, SD—standard deviation, SSD—skin-to-stone distance, MSD—maximum stone diameter, SV—stone volume, MD—mean density of the stone in HU, SMLI—Storz Medical Lithotripsy Index. Statistically significant *p* values are marked in bold.

**Table 3:** Univariate and multivariate logistic regression analysis of the associations between clinical characteristics and treatment success.

Variable	Univariate			Multivariate		
	OR	95% CI	*p* Value	OR	95% CI	*p* Value
SV	0.99	0.99–1.00	**0.012**	1.00	0.99–1.00	0.670
Maximum diameter of stone	0.75	0.62–0.90	**0.002**	0.77	0.52–1.13	0.174
MD	0.99	0.99–1.00	**0.009**	0.99	0.99–1.00	0.176
SSD	0.98	0.95–1.01	0.115	0.98	0.95–1.01	0.250
SMLI/SV ratio	1.58	1.15–2.17	**0.005**	1.06	0.68–1.65	0.811
Sum of SMLI	1.01	1.00–1.01	0.340	1.01	0.99–1.01	0.210

OR—odds ratio, 95% CI—95% confidence intervals, SSD—skin-to-stone distance, MSD—maximum stone diameter, SV—stone volume, MD—mean density of the stone in HU, SMLI—Storz Medical Lithotripsy Index. Statistically significant *p* values are marked in bold.

**Table 4:** Results of the treatment based on Triple D score.

Triple D Score	Successful Treatment	Treatment Failure %
0	33% (2/6)	67% (4/6)
1	48% (12/25)	52% (13/25)
2	76.9% (20/26)	23.1% (6/26)
3	92.9% (13/14)	7.1% (1/14)

## Data Availability

Data is available upon reasonable request.
